# List of Accidents Admitted into the Pennsylvania Hospital, from July 31st to August 14th, 1839, Inclusive

**Published:** 1839-08-24

**Authors:** Henry Wheaton Rivers

**Affiliations:** Resident Surgeon


### CLINICAL REPORTS.

### PENNSYLVANIA HOSPITAL.

*List of Accidents admitted into the Pennsylvania Hospital, from July 31st to August 14th, 1839, inclusive.*

[Reported by HENRY WHEATON RIVERS, M.D., Resident Surgeon.]

A CASE of ununited fracture of both bones of the arm, about their middle. This accident happened about three weeks previous to the patient's entering the hospital, and had been treated in the country during the first two weeks; after which time he came to town, and was under no treatment until he came into the hospital, when it was found that the bones had not united, and that considerable deformity had taken place by their projection inwards. The treatment consisted in applying a compress to the fractured ends of the bones, a splint on the under side of the arm extending to the ends of the fingers, and a bandage firmly applied. The deformity is much less at present, and the patient is doing well.

A case of oblique fracture near the lower end of the radius, caused by a fall on the hand. Treated by a compress over the fracture, two splints, and roller; doing well.

A case of laceration of the hand, by being caught in the machinery of a paper mill; the hand was torn off, leaving nothing but the thumb, which was also badly lacerated. Amputation was performed above the wrist, immediately upon his coming into the house. The ligatures have all come away, and the stump is cicatrizing very well.

A case of lacerated wound of the scalp, with contusion of the left side, from a beating received with a porter bottle. There being considerable hæmorrhage from the wound of the scalp, it was found necessary to use a compress and double-headed roller, which suppressed it effectually. Cold was applied to the head, and six wet cups to side. Discharged cured, five days after the accident.

A case of dislocation of the jaw, caused by yawning, reduced in the usual way.

A case of fracture of the acromion process of the scapula, with partial dislocation of the head of the humerus forwards, from a fall directly upon the back of the shoulder. The dislocation was reduced; but owing to the patient's unwillingness to remain in the house, nothing further was done. The treatment in this case would have been similar to that of fracture of the clavicle.

A case of contusion of the back, with displacement of one or more of the inferior dorsal vertebræ, occasioning paralysis of the lower extremities, bladder, &c.

A case of punctured wound of the finger, from a fish-hook, which was remaining in. It was extracted by first cutting off the flattened end of the shank to which the line is attached, passing the point of the hook through the skin, and drawing it out in that way.

A case of contusion of the leg and ankle, with slight laceration, produced by a fall from a dray. Treated by elevating the limb on an inclined plane, leeches, and cold applications.

A case of punctured wound of the finger, from a fish-hook. This boy was brother to the patient whose case is reported above. The hook was extracted in the same way.

A case of lacerated wound of the scalp, accompanied by one of the right buttock also, caused by a fall from the top of a three story house. The wound of the scalp was dressed with adhesive strips, and cold applications,—and that of the buttock, which was about four inches long, was drawn together with sutures.

A case of compound and comminuted fracture of both bones of both arms, together with a lacerated wound of the scalp, from a fall from a three story window. This patient was a female, æt. sixty-seven, and was subject to fits of somnambulism, under which it was supposed she was labouring at the time of the accident. The scalp was dressed with lint, wet in cold water, the arms put in splints, with the view of making her more comfortable, and a large opiate given. Died twenty-four hours after the accident.

A case of compound dislocation of the second phalanx of the middle finger, which was thrown back upon the first. Reduced by making extension with a narrow, wet bandage, applied by a clove-hitch. Doing well.

A case of incised wound of the wrist, dividing the ulnar artery, and the tendon of the palmaris longus muscle, caused by a cut with a piece of sharp glass; both ends of the divided artery were secured immediately on his coming into the house, together with one other small artery; the parts were brought in apposition by adhesive strips, a splint extending from the elbow to the wound (over which a compress was placed) was applied, and the hand and fingers placed in a flexed position, and retained by means of a roller. Doing well.

A case of oblique fracture of the lower end of the humerus, extending through the internal condyle. This accident happened about ten days before his admission into the hospital, during which time he had received no treatment, and considerable deformity had taken place. The arm was dressed with an angular splint, the angle of which is to be changed every day or two, in order to reduce the deformity as much as possible.

A case of lacerated wound of the hand, caused by a blow from a barrel, which fell upon it. Dressed with a long splint, to prevent motion, and cold applications kept up by means of a syphon, to keep down inflammation.

The amputation of the leg of the boy æt. five, reported in the last number, is now well, the stump having completely cicatrized. The fracture of the clavicle is also well. The case of secondary hæmorrhage, for which the radial artery was taken up, has left the house, the wound having healed.

